# Inflammatory Genetic Markers of Prostate Cancer Risk

**DOI:** 10.3390/cancers2021198

**Published:** 2010-06-08

**Authors:** Elizabeth A. Tindall, Vanessa M. Hayes, Desiree C. Petersen

**Affiliations:** 1 Cancer Genetics Group, Children’s Cancer Institute Australia for Medical Research, Lowy Cancer Research Centre, University of New South Wales, PO Box 81, Randwick, NSW 2031, Australia; E-Mails: etindall@ccia.unsw.edu.au (E.A.T.); vhayes@ccia.unsw.edu.au (V.M.H.); 2 University of New South Wales, Kensington Campus, Sydney, NSW 2052, Australia

**Keywords:** prostate cancer, inflammation, Toll like receptor (TLR), cytokine, chemokine, gene variant, inherited susceptibility

## Abstract

Prostate cancer is the most common cancer in Western society males, with incidence rates predicted to rise with global aging. Etiology of prostate cancer is however poorly understood, while current diagnostic tools can be invasive (digital rectal exam or biopsy) and/or lack specificity for the disease (prostate-specific antigen (PSA) testing). Substantial histological, epidemiological and molecular genetic evidence indicates that inflammation is important in prostate cancer pathogenesis. In this review, we summarize the current status of inflammatory genetic markers influencing susceptibility to prostate cancer. The focus will be on inflammatory cytokines regulating T-helper cell and chemokine homeostasis, together with the Toll-like receptors as key players in the host innate immune system. Although association studies indicating a genetic basis for prostate cancer are presently limited mainly due to lack of replication, larger and more ethnically and clinically defined study populations may help elucidate the true contribution of inflammatory gene variants to prostate cancer risk.

## 1. Introduction

The most recent statistics available from the USA. reveal prostate cancer is now the most frequently diagnosed malignancy in men [1]. World-wide, prostate cancer is the second most commonly diagnosed male malignancy and sixth leading cause of cancer-related male death, equating to a considerable global health burden [2]. Despite this vast prevalence, the number of known risk factors is limited, providing little insight into elucidating which men will be susceptible to developing the disease. The three most significant prostate cancer risk factors to date include increased age, African ancestry and a family history of the disease, with the latter supporting a genetic contribution to prostate cancer risk. Also emerging as a potential mediator of prostate cancer pathogenesis is inflammation. 

**Figure 1:**
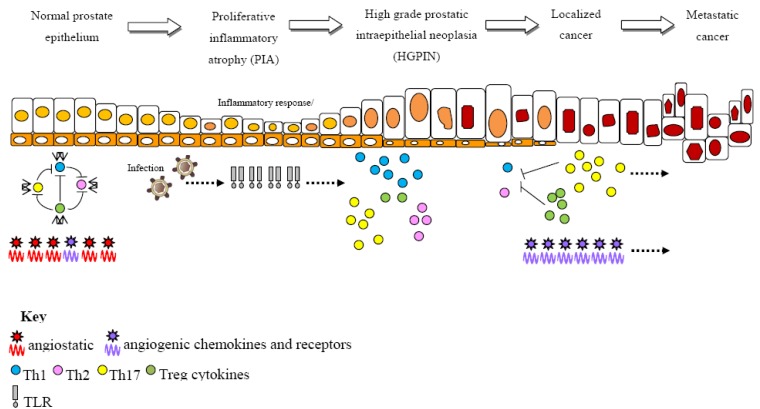
Multi-step process of prostate cancer development. Under normal conditions, Th cytokines are maintained in a homeostatic state via self-regulating mechanisms and angiostatic chemokines are predominant. Regions of PIA are frequently associated with inflammation, possibly triggered by an infectious agent. The inflammatory response induces TLR-expressing inflammatory cells, which mediate cell proliferation and increase cytokine and chemokine production. As the inflammatory response progresses, self-regulating mechanisms fail leading to an overproduction of Treg, Th17 cytokines and angiogenic chemokines, which enhance DNA damage, cell proliferation and angiogenesis, promoting prostate cancer progression.

Despite rising epidemiological evidence linking inflammation and prostate cancer, including an increased risk associated with prior exposure to sexually transmitted infections (STIs) [3,4], the occurrence of clinically diagnosed chronic inflammation of the prostate (prostatitis) [5,6] and an inverse correlation with the use of non-steroidal anti-inflammatory drugs (NSAIDs) [7,8], the precise mechanisms of inflammatory involvement are yet to be determined. In light of a general belief that solid tumor development is a multi-stage process, it has been proposed that regions of chronic inflammation that are coupled with focal atrophy known as proliferative inflammatory atrophy (PIA), may be a pre-cursor to prostate cancer development [9]. The etiology of these lesions, and whether they are likely to act directly by morphing with cancerous cells or indirectly via migration with high grade prostatic intraepithelial neoplasia (HGPIN), are points of debate. Regardless of the etiology (*i.e.*, infectious or non-infectious) of the inflammatory response, chronic inflammation can incite carcinogenesis by inducing proliferative events and post-translational DNA modifications by enhancing the secretion of growth factors such as cytokines and chemokines and inducing oxidative stress by the release of nitric oxide (NO) and reactive oxygen species (ROS) [10]. These inflammatory induced somatic modifications may be responsible for the progression from chronic inflammation of the prostate (prostatitis) to prostate carcinogenesis (Figure 1). 

The inflammatory network is a complex interaction of genes and transcription factors involved in both the relatively non-specific innate immune system and the more targeted adaptive immune system. Mounting an effective immune response relies heavily on a balanced and monitored production of proteins involved in these specific pathways. Given that prostate cancer has been defined as having one of the strongest familial links of all human cancers, it has been hypothesized that genes involved in mediating, inhibiting or maintaining a host immune response may contribute to prostate cancer development and predisposition to prostate cancer risk. 

In this review, we focus on inherited gene variants within key inflammatory genes that have been associated with a varied susceptibility to prostate cancer development. We discuss evidence to support this proposed hypothesis based on genome-wide association studies (GWAS) and family linkage studies, as well as large-scale and more targeted candidate gene analysis. The latter includes the toll-like receptor (TLR) family (innate immune response) and T helper (Th) influencing cytokines and chemokines (adaptive immune response). 

## 2. Non-Targeted Genome-Wide Scans for Prostate Cancer Risk

Methods for unbiased and non-targeted identification of loci contributing to inherited prostate cancer susceptibility include whole genome analysis of linkage in prostate cancer families and large scale case-control association-based approaches. 

One of the first regions to be identified as a potential prostate cancer susceptibility locus using a genome wide scan is on the long arm of chromosome 1 (1q24-25), originally termed hereditary prostate cancer 1 (HPC1) [11]. Analysis of candidate genes within this region identified an anti-viral and anti-proliferative gene involved in the innate immune system, *RNASEL*. In addition to identifying germline mutations within this gene associated with prostate cancer risk, a common variant, R463Q, has been linked to increased susceptibility to a viral infection, namely Xenotropic MulV-related virus (XMRV), proposed to be involved in prostate cancer pathogenesis [12,13]. A second locus identified by familial linkage studies mapped to chromosomal position 8p22, which harbors an innate immune gene, macrophage scavenger receptor 1 (*MSR1*). MSR1 expression is largely restricted to macrophages and is primarily involved in bacterial elimination. Germline variants within this gene have since been associated with prostate cancer risk [14]. The first prostate cancer GWAS to implicate an adaptive cytokine gene variant in prostate cancer risk showed suggestive evidence that the minor allele of a non-synonymous interleukin(*IL*)*-16* variant, rs4072111, may be associated with prostate cancer risk under a recessive model of inheritance [15]. 

Follow-up association studies to confirm a role for variants within the two innate immune genes, *RNASEL* and *MSR1*, in prostate cancer susceptibility, have produced some conflicting results (reviewed in [16]). Similarly, there are no reported studies to verify the association observed for the *IL-16* gene variant and prostate cancer risk. Discrepant results are often achieved for prostate cancer genetic association studies, thus highlighting the complex, heterogeneous nature of the disease and the potential difficulties in determining the genetic contribution to prostate cancer risk. It is proposed that a combination of multiple low- to moderate-risk alleles may contribute to disease risk, although the most appropriate method to accurately define these associations is often disputed.

## 3. Targeted Candidate Gene Analysis for Prostate Cancer Risk

In an effort to clarify a potential role for immune gene variants in prostate cancer risk, one study investigated a large panel of 9,275 variants within 1,086 inflammatory genes using a large and well-defined Swedish case-control population study (CAPS). This study of 400 prostate cancer cases and 400 population-matched controls revealed a multitude of variants across the panel of genes to be significantly associated with prostate cancer risk. By determining that more than the expected number of variants were statistically significant, this report supports previous suggestions and concludes that there is likely an association between prostate cancer risk and multiple modest effect genes within the inflammatory pathways [17]. A targeted approach to investigate specific, candidate inflammatory gene variants might thus provide a suitable means of determining a genetic predisposition to inflammatory mediated prostate cancer. Based on the impact of TLRs, Th cytokines and chemokines in prostate cancer pathogenesis (Figure 1), the gene families encoding these inflammatory agents are addressed herein. 

### 3.1. Toll-like Receptor (TLR) Variants

A host inflammatory response is typically initiated upon recognition of uniquely conserved pathogen associated molecular patterns (PAMPs), generally found on the surface of invading organisms, via pattern recognition receptors (PRRs) of the host innate immune system. The most prominent PRRs are the family of TLRs expressed on the surface of various immune cells. TLRs are a type I transmembrane receptor, of which 13 structurally unique members have thus far been identified. TLRs are characterized by an intracellular toll/IL-1 receptor (TIR) signaling domain and extracellular leucine rich repeats, which determine the recognition of distinct PAMPs. Upon binding to a microbial ligand, TLRs initiate signaling events involved in adaptive immune responses that trigger the production of inflammatory mediators including, cytokines, chemokines, cell adhesion molecules and DNA damaging effector molecules, such as reactive oxygen and nitrogen species [18,19]. TLRs most notably stimulate the production of Th type 1 (Th1), Th17 and CD8^+^ T cell responses, but are also reported to influence Th2 cytokine production under certain pathological conditions [20].

Although the etiology of prostate specific inflammation is currently unknown, it has been suggested that it may evolve in response to pathogenic invasion, which would support a role for TLRs in prostate cancer pathogenesis. Evidence to sustain this hypothesis includes, the observation that bacterial specific RNA is frequently present in cases of clinical prostatitis [21,22], as well as the isolation of infectious agents including human papillomavirus (HPV) and herpes simplex virus (HSV) in prostate cancer tissue [23,24,25]. A more recent report has demonstrated that TLRs, specifically TLR subclass 4 (TLR4) and TLR9, may contribute to prostate cancer pathogenesis by stimulating prostate epithelial cell proliferation in response to infectious stimuli [26]. Polymorphisms that directly influence TLR expression may thus be implicated in a skewed/unregulated inflammatory response to infection, rendering the host more susceptible to prostate carcinogenesis. 

Although a vast number of *TLR* polymorphisms have been identified, to date the functional implications of the majority of these variations are unknown [27]. Regardless, there have been several publications that have investigated a possible association between *TLR* gene polymorphisms and prostate cancer risk, most notably within *TLR4* and the *TLR6*-*TLR1*-*TLR10* gene cluster located within a 54 kb region on chromosome 4p14 (Table 1). Several variant alleles within the *TLR4* gene are reported to be associated with prostate cancer risk, including various genotypes of three polymorphisms, rs1927911, rs10116253 and rs10759932 that have been associated with both an increase [28,29,30] and decrease [31] in prostate cancer risk. Significant associations were also reported for variant alleles within the *TLR6*-*TLR1*-*TLR10* gene cluster, and similarly to *TLR4*, inverse significant associations were observed for alleles at *TLR10* polymorphic sites, rs11096955 and rs11096957 [32,33]. Inconsistent association results are a major concern for disease association studies and often reflect heterogeneity of study design and composition, as well as emphasizing the complex nature of genetic contributions to a multifaceted disease such as prostate cancer. A total of 15 variants across *TLR2*, *TLR3*, *TLR5*, *TLR7*, *TLR8* and *TLR9* have additionally been investigated for association with prostate cancer risk. However to date, no significant associations have been observed for polymorphisms within these genes. 

**Table 1:** TLR variants investigated for association with prostate cancer risk.

Gene family	Gene	Variant ID	^#^ No. of publications	* Population					Ref.	Reported functional effect
			*P* ≤ 0.05	Null	EU	AA	AS	OT		
*TLR*s	*TLR1*	rs4624663		3	√	√	√	√	[32,33,34]	
		rs4833095	1		√	√	√	√	[32]	
		rs5743551	1	2	√	√	√	√	[32,33,34]	
		rs5743556	1	2	√	√	√	√	[32,33,34]	
		rs5743594	1		√	√	√	√	[32]	
		rs5743595	1		√	√	√	√	[32]	√ [35]
		rs5743596	1		√	√	√	√	[32]	
		rs5743604	2	2	√	√	√	√	[32,33,34,36]	
		rs5743611		3	√	√	√	√	[32,33,34]	
	*TLR2*	rs3804100		1	√				[36]	
	*TLR3*	rs3775296		1	√				[36]	
		rs5743305		1	√				[36]	
		rs5743313		1	√				[36]	
	*TLR4*	rs1927911	2	1	√		√		[29,30,31]	
		rs1927914	1	4	√		√		[29,30,31,36,37]	
		rs2149356	1	3	√	√			[28,30,31,37]	
		rs2737190		1	√				[30]	
		rs2770150	1		√				[31]	
		rs4986790	1	4	√	√			[28,30,31,36,37]	√ [38,39]
		rs5030717	1		√				[31]	
		rs5030721		1	√				[36,37]	
		rs5030728	1		√	√			[28]	
		rs6478317	1		√				[31]	
		rs7873784	1	3	√	√			[28,30,31,36]	
		rs10116253	2		√				[30,31]	
		rs10759932	2	2	√	√			[28,31,36,37]	√ [40]
		rs10759933		1	√				[37]	
		rs11536871		2	√				[36,37]	
		rs11536858	1	1	√		√		[29,31]	
		rs11536878		1	√				[31]	
		rs11536889	2	3	√	√			[28,30,31,36,37]	
		rs11536891	1	2	√		√		[29,30,31]	
		rs11536897		2	√		√		[29,31]	
		rs11536898	1	1	√				[30,31]	
	*TLR5*	rs1053954		1	√				[36]	
		rs2072493		1	√				[36]	
		rs5744113		1	√				[36]	
		rs5744174		1	√				[36]	
	*TLR6*	rs1039599		1	√				[34]	
		rs3821985		1	√				[34]	
		rs5743788		2	√				[33,34]	
		rs5743795	1	2	√	√	√	√	[32,33,34]	
		rs5743806	1	2	√	√	√	√	[32,33,34]	
		rs5743810		3	√	√	√	√	[32,33,34]	
		rs5743815		3	√	√	√	√	[32,33,34]	
	*TLR7*	rs179008		1	√				[36]	√ [41]
		rs179019		1	√				[36]	
		rs2302267		1	√				[36]	
	*TLR8*	rs1548731		1	√				[36]	
		rs4830806		1	√				[36]	
		rs5744068		1	√				[36]	
	*TLR9*	rs187084		1	√				[36]	
	*TLR10*	rs4274855	1	2	√	√	√	√	[32,33,34]	
		rs4129009	1	2	√	√	√	√	[32,33,34]	√ [35]
		rs7653908		1	√	√	√	√	[32]	
		rs7658893		1	√	√	√	√	[32]	
		rs10856838		1	√	√	√	√	[32]	
		rs11096955	2	1	√	√	√	√	[32,33,34]	
		rs11096957	2	1	√	√	√	√	[32,33,34]	
		rs11466617	1	2	√	√	√	√	[32,33,34]	
		rs11466640	1	2	√	√	√	√	[32,33,34]	
		rs11466649		1	√	√	√	√	[32]	
		rs11466651		1	√	√	√	√	[32]	
		rs11466653		1	√	√	√	√	[32]	
		rs11466655		1	√	√	√	√	[32]	
		rs11096956		1	√	√	√	√	[32]	
		rs11466657		3	√	√	√	√	[32,33,34]	
	Epistasis									
	*TLR1/ TLR6/ TLR10*	rs11096955/ rs11096957/ rs4833095/ rs5743596/ rs5743595/ rs5743551	1		√	√	√	√	[32]	
		11 SNPs	1		√				[33]	
	*TLR4*	15 SNPs	1		√				[31]	

* Population EU (European), AA (African American), AS (Asian) and OT (Other). # The associations observed include a significance of *P* ≤ 0.05 found with either a specific allele or genotype.

### 3.2. T helper (Th) Cytokine Variants

Th cells form a critical component of the adaptive immune system, though are important contributors to nearly all cellular components of immunity. Unique Th cell phenotypes differentiate from naïve Th cells dependant on the pathogen and subsequent cytokine stimulus present. Several mechanisms are involved in influencing this production and maintaining a balanced and effective Th cell response, most notably via self-regulating mechanisms of Th secreted cytokines. Disruption at any of these check points may lead to auto-immune and other chronic inflammatory disorders. 

A role for Th cells in prostate cancer pathogenesis is becoming increasingly evident. In addition to being associated with an improved response to prostate cancer immunotherapy [42], Th cells have been negatively implicated in prostate cancer pathogenesis [43], demonstrating pleiotropic roles in prostate cancer immunity. More specific phenotypic analysis of prostate infiltrating lymphocytes (PILs) in the tumor microenvironment has indicated that a significant portion of the cells, which infiltrate the cancerous prostate gland, are skewed towards a Th17 or possibly a FoxP3 expressing T regulatory (Treg) cell type [44]. In further support of a role for Th17 cells in prostate cancer, investigators have recently reported an inverse relationship between the frequency of circulating Th17 cells and time till progression to metastatic disease [45]. Although this work is still relatively novel and incomplete, elucidation of the effector cell subtypes likely involved in prostate cancer development provides a strong basis for targeting specific cytokines involved in maintaining a Th cell balance for association with prostate cancer risk.

Genetic variation within genes that encode cytokines involved in regulating a Th cell balance may alter the nature and extent of an adaptive immune response, thus predisposing individuals to an increased risk of inflammatory mediated prostate cancer. Since this hypothesis was proposed, numerous studies have investigated genetic associations between Th cytokine variants and prostate cancer risk. As is typically observed for prostate cancer, the literature surrounding this hypothesis is somewhat controversial, with several association studies reporting conflicting results. Although numerous attempts have been published, significant associations to date are limited to variants within the Th influencing cytokines, *IL-1β* [46], *IL-2* [47], *IL-6* [48,49,50], *IL-10* [30,46,51,52,53,54], *IL-18* [55], transforming growth factor (*TGF*)-*β1* [56,57,58,59] and tumor necrosis factor (*TNF*) [60,61] (Table 1). Data generated in our laboratory additionally supports a role for gene variants within cytokine encoding genes *IL-4* and *IL-6* and susceptibility to prostate cancer. Amongst the significantly associated variants, functional studies have implicated *IL-10* rs1800896 (-1082G>A) [62], *IL-18* rs187238 (-137G>C) [63], *IL-6* rs1800795 (-174G>C) [64,65,66,67,68], *TGF*-*β1* rs1800469 (-509C>T) [69,70,71] and rs1800470 (896T>C, Leu10Pro) [72,73,74] and *TNF* rs1799724 (-857C>T) [75] and rs1800629 (-308G>A) [76,77] in differential gene activity. Reports of functional significance for variants observed to be associated with prostate cancer risk further supports the possibility that Th variants may play a role in inflammatory induced prostate cancer by skewing the Th cell balance from its homeostatic state (Figure 1). A direct functional role for Th cytokines in prostate cancer pathogenesis provides a basis for targeting this pathway in developing prostate cancer immunotherapies. In conjunction with reports that implicate Th cytokine gene variants in prostate cancer risk, numerous studies have failed to report an association, including some reports which refute previous associations (summarized in Table 2).

**Table 2:** Th cytokine variants investigated for association with prostate cancer risk.

Gene familyGene Variant ID			^#^ No. of publications		* Population				Reference	Reported functional effect
			*P* ≤ 0.05	Null	EU	AA	AS	OT		
**Th Cytokines**	*IL-1α*	rs1800587		1	√				[61]	√ [78]
	*IL-1β*	rs1143627		3	√				[30]	√ [79,80]
		rs16944		4	√	√			[46,53,54,81]	√ [82,83]
		rs1143634	1	2	√	√			[46,54,81]	
	*IL-1RN*	rs878972		1	√				[84]	
		rs315934		1	√				[84]	
		rs3087263		2	√	√			[84,85]	
		rs380092		1	√	√			[85]	
		rs4252019		1	√	√			[85]	
		rs579543		1	√	√			[85]	
		rs315951		2	√	√			[84,85]	
		rs4252041		1	√	√			[85]	
		rs9005		1	√	√			[85]	
	*IL-2*	rs2069762		1	√				[86]	√ [87,88]
		rs2069763	1				√		[47]	
		rs3136534		1	√				[86]	
	*IL-4*	Intron 3, 70bp VNTR		1				√	[48]	√ [89]
	*IL-6*	rs1800797		3	√	√			[30,46,90]	
		rs1800796		3	√	√			[30,90,91]	√ [66,92]
		rs1800795	3	6	√	√		√	[30,46,48,49,50,81,90,91]	√ [64,65,66,67,68]
		rs2069830		1		√			[91]	
		rs2069832		2	√	√			[46,49]	
		rs1474348		1	√				[90]	
		rs2069837		2	√	√			[90,91]	
		rs2069860		1	√				[90]	
		rs1474347		1		√			[91]	
		rs1524107		1		√			[91]	
		rs1554606		1	√	√			[91]	
		rs2069849		2	√	√			[49,91]	
		rs1818879		1	√	√			[91]	
	*IL-10*	rs1800896	5	3	√	√		√	[30,46,51,52,53,54,81]	√ [62]
		rs1800871	2	3	√	√		√	[46,51,52,54,81]	
		rs1800872	2	3	√	√			[30,46,51,54,93]	
		rs3024496		2	√	√			[51,81]	
	*IL-18*	rs1946518		1			√		[55]	√ [63]
		rs187238	1				√		[55]	√ [63]
	*IL-21*	rs6822844		1	√				[86]	
		rs6840978		1	√				[86]	
	*TGF-β1*	rs1800468		1	√	√			[94]	√ [69]
		rs1800469	2	1	√	√			[56,57,94]	√ [69,70,71]
		rs1800470	2	3	√	√	√		[57,58,59,95,94,95,94,95]	√ [72,73,74]
		rs1800471		1	√	√			[94]	√ [96]
		rs1800472		1	√	√			[94]	
	*TNF*	rs1799964	1	1	√			√	[60,97]	
		rs1800630		2	√			√	[60,97]	
		rs1799724	1	3	√	√		√	[46,54,60,97]	√ [75]
		rs1800629	1	6	√	√		√	[30,46,49,54,60,61,97]	√ [76,77,98]
		rs361525		3	√	√			[46,54,97]	√ [99]
		rs3093661		1	√				[49]	
		rs1800610		1	√				[97]	
		rs3093668		1	√				[49]	
	Epistasis									
	*IL-1β/IL-10*	rs1143627/ rs1800896	1		√				[46]	
		rs1143627/ rs1800896	1		√				[54]	
		rs16944/ rs1800872	1			√			[54]	
	*IL-1RN*	rs878972/ rs315934/ rs3087263/ rs315951	1		√				[84]	
	*IL-10/TNF*	rs1800872/ rs361525	1		√				[54]	
	*IL-10*	rs1800896/ rs1800871/ rs1800872/ rs3024496	1		√				[51]	√ [62,100,101]
		rs1800896/ rs1800871	1					√		
	*IL-18*	rs1946518/ rs187238	1				√		[55]	√ [63]
	*TNF*	rs1799964/ rs1800630/ rs1799724/ rs1800629	2		√			√	[60,97]	

* Population EU (European), AA (African American), AS (Asian) and OT (Other). # The associations observed include a significance of *P* ≤ 0.05 found with either a specific allele or genotype.

### 3.3. Chemokine variants

The largest subset of cytokines is known as the chemokine network, which comprises small (8–10 kDa) chemoattractant proteins referred to as chemokines (approximately 50) that serve as ligands for G-protein-coupled seven-transmembrane domain chemokine receptors (approximately 20). Chemokines and their receptors are grouped into four classes (C, CC, CXC and CX3C) based on the position of two of four highly conserved cysteine residues near the amino terminus of the protein. Activation of the chemokine network is essential for the regulation of signaling cascades that induce cell migration to specific sites during inflammation. 

Tumor cells, including prostate cancer cells, have been found to express chemokines and their receptors, which act as growth or survival factors, regulate angiogenesis, determine metastatic spread and control leukocyte infiltration into tumors, thereby hindering antitumor immune responses [102]. CXC chemokines containing a Glu-Leu-Arg/ELR+ motif preceding the first cysteine residue are considered to be angiogenic (CXCL1, 2, 3, 5, 6, 7 and 8), while CXC chemokines lacking this ELR- motif are angiostatic (CXCL4, 9, 10, 11, 14). However, it has been observed that although CXCL12 is an ELR- CXC chemokine, it has been found to be angiogenic [103,104]. Various studies have indicated that normal prostate epithelium produces relatively high levels of angiostatic chemokines and low levels of angiogenic chemokines [105,106,107]. To date, research on prostate cancer and the chemokine network has mainly focused on CCL2, CXCL8 and CXCL12 and their receptors [106]. Many other chemokines and their receptors have also been shown to have multifaceted roles in the progression of prostate cancer [106,107,108]. 

The chemokine network regulates inflammatory responses and clearly contributes to inflammatory mediated prostate cancer [106,107,108]. It has been further suggested that chemokines and their receptors may also play a role in the variable incidence rates observed for prostate cancer as their expression profiles appear specific for certain populations affected by the disease [106]. Genes encoding chemokine and chemokine receptors may therefore contain genetic markers that influence prostate cancer risk. Although genetic variation in the chemokine network has been associated with influencing susceptibility to a number of cancers, studies have been limited for prostate cancer (Table 1). A functional polymorphism in the promoter region of *CXCL8* (*IL8)*, rs4073 (-251A/T), has been previously associated with prostate cancer risk. More specifically, the rs4073 TT genotype (low producer of CXCL8) was associated with decreased risk for developing prostate cancer [53], although this finding was not replicated in another study with an increased sample size [109]. In addition, the latter study did not show any association between two *CXCL8* receptor gene polymorphisms, *CXCR1* rs2230054 (860C>G) and *CXCR2* rs11226580 (-1010A>G), and prostate cancer risk [109]. The *CXCL12* rs1801157 variant has been shown to influence the development of prostate cancer with the GA and AA genotypes (increased production of *CXCL12*) being associated with an increased disease risk in a Japanese sample population [110].In contrast, our group found no significant association for the *CXCL12* polymorphism with risk status in our larger Australian case-control population-based study [111]. Using the same study population, commonly investigated *CCL5* rs2107538 (-403G>A), *CCR2* rs1799864 (G>A; V64I), *CCR5* rs333 (Δ32), *CX3CR1* rs3732379 (G>A; V249I) and rs3732378 (C>T; T280M) functional variants were also found to be insignificant contributors to prostate cancer susceptibility [111]. An association was however observed between the *CCR5*Δ32 marker, rs333, (no production of CCR5) and familial prostate cancer risk when considering the number of first-degree relatives of cases who are affected with prostate cancer [111]. Recently, a small study consisting of 50 centenarians as controls with population matched cases reported rs333 as having a protective effect against the development of prostate cancer [112]. These discrepant associations require replication in independent family and case-control studies (Table 3).

**Table 3:** Chemokine variants investigated for association with prostate cancer risk.

Gene family	Gene	Variant ID	^#^ No. of publications		* Population				Ref.	Reported functional effect
			*P* ≤ 0.05	Null	EU	AA	AS	OT		
**Chemokines**	*CCL2* (*MCP1*)	rs1024611		1	√				[61]	√ [113]
	*CCL5* (*RANTES*)	rs2107538	1	1	√				[61,111]	√ [114]
	*CCR5*	rs333	1	1	√				[111,112]	√ [115]
	*CCR2*	rs1799864		1	√				[111]	√ [116]
	*CXCL8* (*IL-8*)	rs4073	1	1	√				[53,109]	√ [117]
	*CXCL12* (*SDF1*)	rs1801157	1	1	√		√		[110,111]	√ [118]
	*CXCR1*	rs2230054		1	√				[109]	
	*CXCR2*	rs11226580		1	√				[109]	
	*CX3CR1*	rs3732378		1	√				[111]	√ [119]
		rs3732379		1	√				[111]	√ [119]

* Population EU (European), AA (African American), AS (Asian) and OT (Other). # The associations observed include a significance of *P* ≤ 0.05 found with either a specific allele or genotype.

## 4. Conclusions

Although evidence is accumulating on the importance of inflammation in prostate cancer etiology, genetic association studies investigating the role of immune related gene variants in susceptibility to prostate cancer are still in their infancy. In addition to describing evidence of association, this review details some of the disparities between association results for TLR, Th cytokine and chemokine gene variants and prostate cancer risk. Failure to replicate association results is not unique to prostate cancer, but is rather a wide-spread issue particularly related to this candidate gene approach for genetic association studies. Some major factors contributing to the inconsistency are related to study design (e.g., case control *versus* case cohort), genotyping methodology and error rate, sample size and ethnic diversity, recruitment strategy and interpretation of results (*i.e.*, adequate *P* value and correction for multiple testing) [120]. Together with the lack of available data and discrepancies reported, we call for a more focused research effort, which may include using larger, more stringently defined (in terms of clinical/pathological features and ethnicity) population-based studies to investigate the complex network of inflammatory genes as markers of risk for developing prostate cancer.

We have mentioned frequently throughout this review that prostate cancer association studies should be assessed in ethnically unique population groups to account for the racial discrepancies which exist for prostate cancer risk. African Americans are the highest known risk group, while Asians have the lowest attributable risk [121]. When segregating ethnic groups for association analysis, population stratification must be adjusted for as subpopulations will influence variant selection and the outcome of the association analysis [122]. 

Distinct from TLR cytokine variants, where there generally appeared to be no difference in disease risk between localized and advanced prostate cancer cases [29,31,32,34,37] (indicative of genetic variation influencing disease development rather than progression), it is important to note that several of the positively associated Th cytokine variants discussed in this review have been associated with specific disease characteristics including, prostate cancer grade and metastasis [46,48,50,51,52,56,57,60]. Subsequently, one hypothesis suggests that if various components of the host immune system appear implicated in different stages of disease progression (Figure 1), the effect of immune related variants may be specific to certain clinicopathological characteristics of prostate cancer. Elucidating true associations may thus require large cohorts of more clinically and geographically defined subsets of prostate cancer.

The complexity of genetic involvement in prostate cancer pathogenesis is highlighted by recent GWAS (predominantly European-based) that have described numerous allelic variants located at several distinct regions across the genome, to have a moderate effect on prostate cancer risk (reviewed in [123]). Prostate cancer therefore has among the highest number of identified risk loci of any disease studied to date. Although inflammatory gene variants are presently not among these risk alleles, the modest contribution of current genetic risk factors is not adequate to explain the familial risk previously described for prostate cancer. Thus, the identification of risk alleles is likely to grow, aided by the collection of ethnically diverse study populations and the expansion of known genetic content.

The modest effect described for the vast majority of significantly associated variant alleles, has contributed to the consensus that prostate cancer fits into the common disease, common variant hypothesis, which implies that multiple variants are likely responsible for the observed inherited disease risk [124]. Inflammation involves a complex interaction of gene networks and is largely self-regulating, thus it is reasonable to assume that certain combinations of alleles may contribute to an imbalanced immune response and increased prostate cancer risk. We have described here some instances of epistasis contributing to prostate cancer risk, which implies these variant alleles act synergistically to effect disease susceptibility. Given the complex nature of prostate cancer and the likely possibility that multiple gene variants are involved, epistasic interactions between inflammatory gene variants and also gene-environment interactions are worthy of further investigation. 

Identification of gene variants responsible for the inherited component of prostate cancer risk may contribute to the development of genetic based screening tests to assist current diagnostic strategies, facilitate defining what is essentially a heterogenous disease and more accurately identify men in the community at a greater risk of developing the disease. In addition to improving clinical management, such findings could provide novel targets for the intervention of prostate cancer therapies.

Current treatment options available for prostate cancer can be both invasive and toxic, negatively impacting on quality of life and prompting many patients (particularly those in the later stages of life) to refuse treatment. The primary goal for developing immunotherapeutics for prostate cancer is to prevent disease progression, whilst providing a cancer specific treatment that minimizes toxicity and other detrimental side-effects. Currently there are no approved immunotherapeutic treatments for prostate cancer, however advances in the field of immunology has provided a better understanding of antigen presentation, antigen recognition and tumor immune escape, making the task more feasible. Identification of variants responsible for maintaining a tumor immune response may therefore further provide more specific targets to combat the development of prostate cancer and disease progression.
